# Targeting Thioredoxin Reductase 1 Reduction in Cancer Cells Inhibits Self-Sufficient Growth and DNA Replication

**DOI:** 10.1371/journal.pone.0001112

**Published:** 2007-10-31

**Authors:** Min-Hyuk Yoo, Xue-Ming Xu, Bradley A. Carlson, Andrew D. Patterson, Vadim N. Gladyshev, Dolph L. Hatfield

**Affiliations:** 1 Molecular Biology of Selenium Section, Laboratory of Cancer Prevention, Center for Cancer Research, National Cancer Institute, National Institutes of Health, Bethesda, Maryland, United States of America; 2 Department of Biochemistry, University of Nebraska, Lincoln, Nebraska, United States of America; Ordway Research Institute, United States of America

## Abstract

Thioredoxin reductase 1 (TR1) is a major redox regulator in mammalian cells. As an important antioxidant selenoprotein, TR1 is thought to participate in cancer prevention, but is also known to be over-expressed in many cancer cells. Numerous cancer drugs inhibit TR1, and this protein has been proposed as a target for cancer therapy. We previously reported that reduction of TR1 levels in cancer cells reversed many malignant characteristics suggesting that deficiency in TR1 function is antitumorigenic. The molecular basis for TR1's role in cancer development, however, is not understood. Herein, we found that, among selenoproteins, TR1 is uniquely overexpressed in cancer cells and its knockdown in a mouse cancer cell line driven by oncogenic *k-ras* resulted in morphological changes characteristic of parental (normal) cells, without significant effect on cell growth under normal growth conditions. When grown in serum-deficient medium, TR1 deficient cancer cells lose self-sufficiency of growth, manifest a defective progression in their S phase and a decreased expression of DNA polymerase α, an enzyme important in DNA replication. These observations provide evidence that TR1 is critical for self-sufficiency in growth signals of malignant cells, that TR1 acts largely as a pro-cancer protein and it is indeed a primary target in cancer therapy.

## Introduction

Dietary selenium has potent cancer prevention activity [1] and both selenium-containing proteins (selenoproteins) [2,3 and references therein] and low molecular weight selenium-containing compounds (selenocompounds) [2 and references therein] have been implicated in this activity. The major role of selenium in providing health benefits is likely through the action of selenoproteins [1]. Thioredoxin reductase 1 (TR1) is one of 24 known selenoproteins in rodents [4], is a major antioxidant and redox regulator in mammalian cells [5,6 and references therein] and has an essential role in mammalian development [7]. However, this enzyme appears to have opposing effects in cancer development as it has been implicated in both cancer prevention [8] and cancer promotion [9]–[12]. For example, TR1 supports p53 function and has other tumor suppressor activities, and its targeting by carcinogenic, electrophilic compounds argue for its role in cancer prevention [13]. Alternatively, TR1 is overexpressed in many cancer cells [9]–[12] and its inhibition by a variety of potent cancer drugs altered cancer-related properties of numerous tumors and malignant cells suggesting that this enzyme is a target for cancer therapy [9]–[12], [14], [15].

It is not clear whether TR1 cancer-preventing or cancer-promoting properties exert greater influence on cancer, whether these contrasting effects operate simultaneously or are specific to different stages of cancer development, and how these properties could be utilized in cancer prevention and/or therapy. To address these issues, we initially examined the role of TR1 in a mouse lung cancer cell line and a mouse animal model and observed that reduction of TR1 levels reversed numerous malignant properties including tumorigenecity [16]. Herein, we examined TR1 function and roles in cancer development in a malignant mouse cell line for which the corresponding parental (normal) cell line was available, and further verified the findings in several human cancer cell lines.

## Results

### Generation of the malignant and parental cell lines and analysis of their TR1 levels along with several cancerous human cell lines

**Figure 1:**
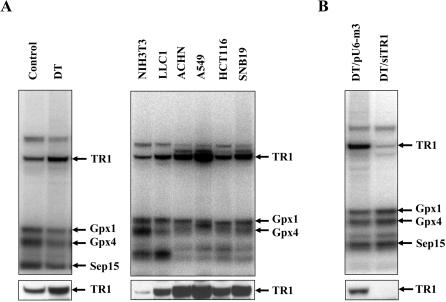
Thioredoxin reductase (TR1) expression in mouse and human malignant cells. Indicated cell lines were metabolically labeled with ^75^Se, the resulting protein cell extracts electrophoresed and the gels exposed to a PhosphorImager (see Methods). Selenoproteins identified previously in cell extracts [25], [26] are indicated by an arrow and name on the right of each panel. TR1 was also identified by western blotting in protein extracts of each cell line as shown in the lower panels. (A, left panel) Control (parental; NIH3T3) and DT cells. (A, right panel) NIH3T3 (control, parental cells used in generating DT cells), LLC1 (mouse Lewis lung cell carcinoma), ACHN (human kidney renal cell carcinoma), A549 (human lung non-small cell carcinoma), HCT116 (human colon cell adenocarcinoma) and SNB19 (human cell glioblastoma). NIH3T3 was used as an indicator cell line for comparison of TR1 levels in a normal cell line to the malignant cell lines. (B) DT/pU6-m3 and DT/siTR1. DT (obtained by overexpression of mutant *k-ras* in NIH3T3) cells were stably transfected with the pU6-m3 (control) vector or siTR1 knockdown vector, respectively (see Methods).

DT cells, which encode oncogenic *k-ras* [17], [18], and the parental NIH3T3 (control) cells were labeled with ^75^Se and the resulting protein extracts electrophoresed to examine the levels of TR1 and other selenoproteins (Fig. 1A, upper left panel). The 55 kDa TR1 is one of the major selenoproteins, along with other selenoproteins, labeled in this figure. Clearly, DT cells have higher amounts of TR1 than control cells. TR1 levels were also examined in control and DT cells by western blotting (Fig. 1A, lower left panel) which confirmed increased levels of TR1 in DT cells. Interestingly, elevated TR1 expression was at the expense of other selenoproteins, which were at reduced levels in DT cells compared to control cells.

In addition, mouse Lewis lung carcinoma (LLC1) cells [16] along with four human cell lines, along with, were labeled with ^75^Se and selenoprotein expression analyzed (Fig. 1A, right upper panel). Although no corresponding control cells were available for these malignant cell lines, we compared their selenoprotein labeling patterns to those of normal NIH3T3 cells. The only labeled selenoprotein that appeared to be overexpressed in each of the cancer cells lines was TR1. Western blot analysis also showed high levels of TR1 in the human and mouse malignant cell lines (Fig. 1A, lower right panel).

### Stable transfection of DT and control cells with a siRNA-TR1 knockdown vector and their characterization

DT cells were stably transfected with the siRNA-TR1 knockdown (designated DT/siRNA) or control vector (designated DT/pU6-m3) (Fig. 1B, upper panel). TR1 was virtually absent in DT/siRNA transfected cells as demonstrated by metabolic ^75^Se labeling and western blotting (Fig. 1B, lower panel).

**Figure 2:**
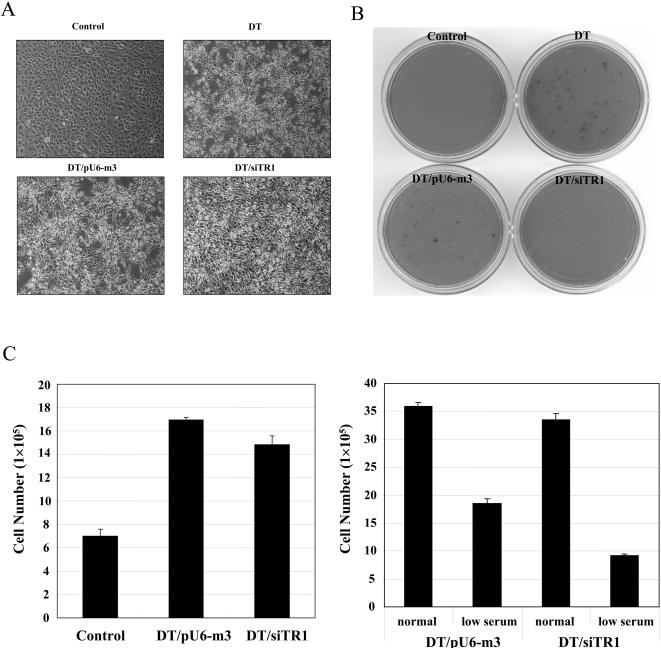
Morphology, growth in soft agar and growth rates of TR1-expressing and -deficient cells. (A) Control (NIH3T3, parental), DT, DT/pU6-m3 and DT/siTR1 cells. Cells were grown on culture plates and photographed during exponential growth (see Methods). (B) Anchorage-independent growth of control, DT, DT/pU6-m3 and DT/siTR1 knockdown cells. One thousand cells were suspended in soft agar and grown for two weeks. Plates were then stained with INT overnight and photographed. Details are given in Methods. (C) Growth rates of control, DT/pU6-m3 and DT/siTR1 cells under normal and serum-deficient conditions. Control, DT/pU6-m3 and DT/siTR1 cells were seeded (2×10^5 ^cells/60mm dish) and grown under normal growth conditions (left panel), and DT/pU6-m3 and DT/siTR1 cells were seeded (5×10^5 ^cells/60 mm dish) and grown in serum-deficient medium (right panel). Growth rates in serum-deficient medium were compared to those obtained under normal growth conditions.

The phenotypes of the two transfected DT cell lines were examined and compared to those of untransfected DT and control cells (Fig. 2). Control cells grew in monolayer and tightly attached to the culture dish which are characteristics of normal cells, while DT/pU6-m3 and DT cells grew in multilayer and loosely attached to the culture dish which are characteristics of malignant cells (Fig. 2A). However, DT/siRNA cells had a significantly diminished ability to grow in multilayer and were more tightly attached to the culture dish than either DT/pU6-m3 or DT cells.

Since many cancer cells can grow unanchored in soft agar, but most normal cells cannot, the ability of control, DT, DT/pU6-m3 and DT/siTR1 cells to grow in soft agar was examined (Fig. 2B). DT and DT/pU6-m3 cells grew in soft agar, while control and DT/siTR1 cells grew poorly under these conditions. The growth properties in soft agar of two human cell lines, A549 and HCT116, following transfection with the corresponding human TR1 knockdown vector and control vector lacking siTR1 were also examined (see Fig. S1 and figure legend). Interestingly, both cell lines encoding siTR1 lost their ability to grow in soft agar.

### Self-sufficiency in growth signals

Self-sufficiency in growth signals has been suggested as one of six acquired capabilities of cancer phenotypes and the Ras-Raf-MAPK signaling cascade is known to be involved in this acquired capability by mimicking growth signals [19]. We examined the effect of TR1 reduction on this phenotype by growing control, DT/pU6-m3 and TR1 knockdown cells in regular and serum-deficient media (Fig. 2C). Under normal growth conditions, both DT and DT/siTR1 cells grew more than twice the rate of NIH3T3 cells, while DT/siTR1 cells grew only about 10% less effectively than DT/pU6-m3 cells (left panel, Fig. 2C). Thus, TR1 knockdown did not decrease the growth of DT cells, excluding the effects associated with TR1 essentiality for cell growth. As many malignant cells grow more efficiently than normal cells when cultured in serum-deficient medium, we examined the abilities of the two transfected cell lines to grow in serum-deficient conditions (right panel). DT/siTR1 cells grew at about half the rate as the DT/pU6-m3 cells, further suggesting that cancer-associated properties of cells were specifically affected by the TR1 knockdown.

### Cell cycle analsis

**Figure 3:**
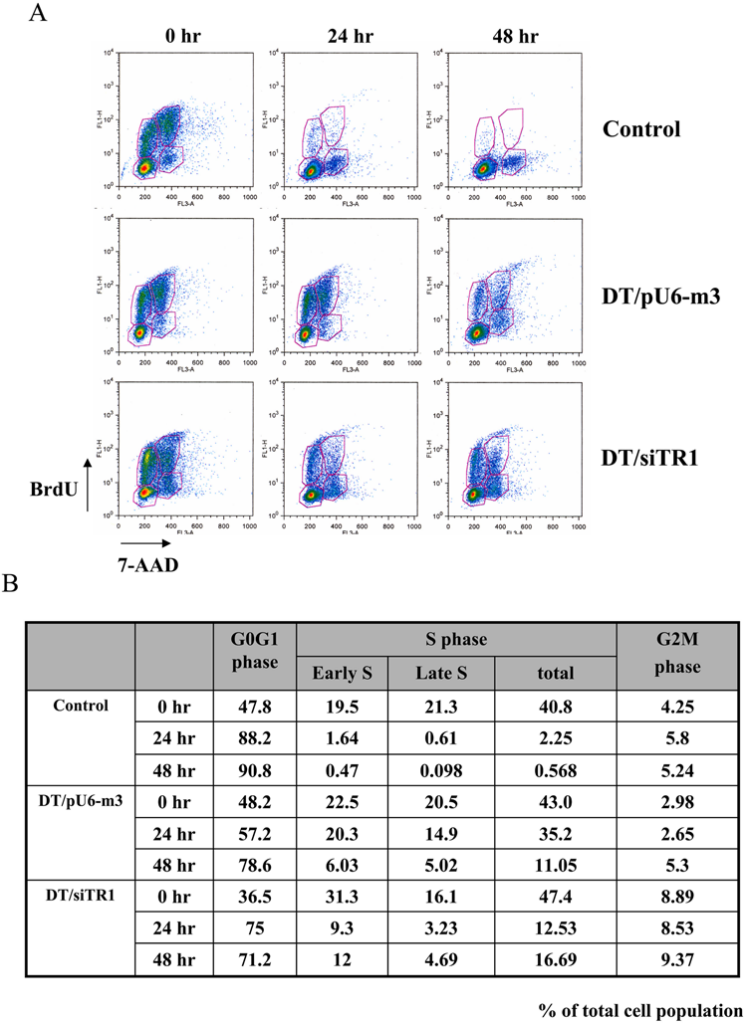
Cell cycle analysis. Control, DT/pU6-m3 and DT/siTR1 cells were grown in serum-deficient medium for 0, 24 and 48 hrs and incubated with BrdU for 6 hours. Harvested cells were stained with anti-BrdU antibody to monitor newly replicated genomic DNA and 7-AAD to monitor whole genomic DNA. (A) Stained cells were analyzed by flow cytometry and (B) quantitated in each phase of the growth cycle by FlowJO. Phases of the growth cycle, G0-G1, S phase (Early S and Late S) and G2-M were shown and the values given represent the percent of the total cell population. Details of the experiments shown in the figure are given in Methods.

Cell cycle analysis of control, DT/siTR1 and DT/pU6-m3 cells, when grown in serum-deficient medium, was carried out to elucidate the stage affected by the reduction in TR1 expression resulting in growth retardation (Fig. 3). The three cell lines were grown in complete medium overnight and then placed in serum-deficient medium for 0, 24 and 48 hrs. Cells were incubated with 5-bromo-2-deoxyuridine (BrdU) and stained with BrdU antibodies to assess DNA replication and were incubated with 7-amino-actinomycin (7-AAD) to assess genomic DNA. These cells were then analyzed by FACS (Fig. 3A) and the appropriate areas quantitated (Fig. 3B). The three cell lines had large numbers of cells in both the G0-G1 and S phases when analyzed immediately following active growth in complete medium, although DT/siTR1 had more cells in the S phase while the other two cell lines had more cells in the G0-G1 phase (see Time 0 in Figs. 3A and B). The control cell line had approximately 90% of its cells retained in the G0-G1 phase throughout growth in serum-deficient medium. This observation most certainly indicates that most of the control cells are retained in the quiescent (G0) state which is consistent with their inability to grow under these growth conditions. A major difference occurred in the amounts of cells in DT/pU6-m3 and DT/siTR1 cell lines at 24 hrs of growth in the G0-G1 and S phases in that DT/pU6-m3 cells had a much higher percentage of its cells in the S phase and lower percentage in the G0-G1 phase than did DT/siTR1. Within the S phase, DT/pU6-m3 had about 1.5 times more cells in early than late S, while DT/siTR1 had about 3 times more cells in early than late S. Although the amounts of cells in both transfected cell lines in the G0-G1 and S phases more closely approximated each other at 48 hrs of growth, the large divergence was still present between the early and late S phases in these two cell lines. These findings suggest that DNA replication was arrested in the early S phase in DT/siTR1 cells. It should also be noted that DT/siTR1 cells have higher amounts of their cell population retained in the G2-M phase than either control or DT/pU6-m3 cells during growth analysis suggesting that more cells within the DT/siTR1 population have trouble transitioning into mitosis. The possible defect causing greater retardation of DTsiTR1 cells in the G2-M phase warrants further investigation but was not pursued in this study as it does not appear to affect the overall reduction in DT/siTR1 growth rate compared to that of DT/pU6-m3 in serum-deficient medium (see Fig. 2C).

### Analysis of DNA polymerase factors

**Figure 4:**
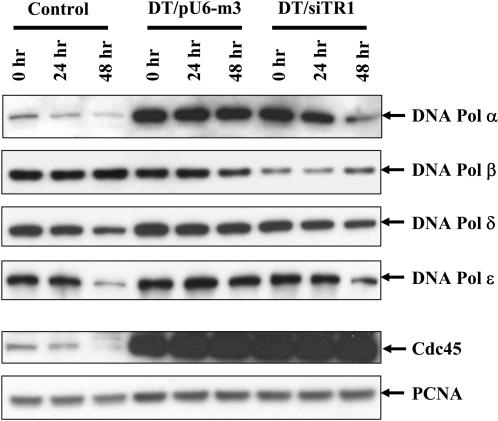
Analysis of components involved in DNA replication. Expression levels of DNA polymerase components, DNA Pol α, β, δ and ε, Cdc45 and PCNA, in control, DT/pU6-m3 and DT/siTR1 cells were analyzed by western blotting. Cells were grown in serum-deficient medium for 0, 24 and 48 hrs, harvested, protein cell extracts prepared and electrophoresed as described in Methods.

To elucidate which component(s) might be involved in DNA replication causing a reduction in overall growth rate in serum-deficient medium in DT/siTR1 cells compared to DT/pU6-m3 cells, we examined the levels of DNA Pol α, β, γ and ε, Cdc45 and PCNA which all play a role in this process [20]. Western analyses of these components were carried out with the three cell lines at each time point (Fig. 4). DNA Pol α and Cdc45 were highly enriched in both transfected cell lines compared to control cells at each of the time points. Interestingly, DNA Pol α appeared to be reduced in DT/siTR1 cells compared to DT/pU6-m3 cells at 24 hrs and most significantly at 48 hrs. In addition, the levels of DNA Pol ε appeared to be reduced in control and DT/siTR1 cells compared to DT/pU6-m3 cells at 48 hrs, while DNA Pol β appeared to be reduced at all three time points in DT/siTR1 compared to either control or DT/pU6-m3 cells. It would seem that the most pronounced effect of TR1 reduction in DT/siTR1 cells is on DNA Pol α resulting in reduced growth of these cells in serum-deficient medium. However, TR1 reduction may also result in reducing the levels of DNA Pol β, whereas the reduced levels of DNA Pol ε at 48 hrs in control and DT/siTR1 cells may result from the serum-deficient growth medium.

## Discussion

TR1 has many cellular functions and is broadly involved in cellular processes that are regulated by redox [9]–[12]. For example, TR1 has roles in cell proliferation, angiogenesis, transcription, DNA repair and serves in antioxidant defense and redox regulation of cell signaling (see reviews [9]–[12]). Its major function is thought to maintain cytosolic thioredoxin (Trx1) in the reduced state using NADPH as an electron donor [21]. In turn, Trx1 donates reducing equivalents to disulfides in nuclear and cytosolic proteins maintaining reduced cysteine residues in these proteins. Herein, we elucidated the role of TR1 in self-sufficiency of growth by showing that inhibition of DNA replication in a malignant TR1 knockdown cell line is associated with a reduction in DNA Pol α expression. In addition, other studies have suggested that DNA replication can be retarded by limiting the DNA synthesis since thioredoxin has a role in maintaining the intracellular deoxynucleotide pool [11], [22], [23]. However, we ruled out this possibility by examining the deoxynucleotide pools in control, DT, DT/pU6-m3 and DT/siTR1 cells and showing that they did not vary during growth in serum-deficient medium (see Fig. S2).

Numerous TR1 functions and properties suggest that this selenoprotein has anti-cancer functions: 1) oxidative stress is one of major characteristics of cancer cells and TR1 is a key player in antioxidant defense by reducing Trx1 and other redox regulators in cells [9]–[12]; 2) TR1 is essential for proper function of tumor suppressor p53 [13]; 3) TR1 inhibition by carcinogenetic, electrophilic compounds implicated it in cancer prevention [1]; and 4) the selenium-containing amino acid, Sec, occupies the active catalytic site of TR1 [4] and selenium is known to have potent cancer prevention properties [1]. However, TR1 has also been implicated in tumor development and maintenance: 1) it is overexpressed in many tumors and cell lines [9]–[12]; 2) several antitumor drugs are potent inhibitors of TR1 suggesting that this enzyme is a target for cancer therapy [9]–[14]; 3) the targeted removal of TR1 reverses morphology and other cancer characteristics of malignant cells [16] (see also Figs. 2 and 3 and Text S1); and 4) selenium deficiency has been reported to decrease tumor formation is some cancer models in animals [24].

How can the role of TR1 in cancer development be reconciled with its role in tumor suppression as well as the known anti-cancer properties of selenium, which is a catalytic component of TR1? It is tempting to speculate that adequate amounts of dietary selenium in general, and a sufficient expression level of TR1 in particular, maintain cellular redox homeostasis in normal cells, protecting them against oxidative stress, mutations in DNA and damage to other cellular components. Thus, TR1, along with other selenoproteins, can function in cancer prevention by inhibiting malignant transformation. However, in newly emerging tumors, demands for TR1 greatly increase as this protein would seem to be required to sustain tumor growth, likely because of the increased demand for its reducing equivalents via the Trx1 pathway and, as shown in this work, DNA replication. This proposal explains both the potent cancer prevention activity of dietary selenium and the contrasting roles of TR1 in both preventing and promoting cancer. Furthermore, this study provides the basis to explain disparate literature data on the role of this enigmatic protein in cancer and elevates TR1 even further as a [8]–[12], [14]–[16] will undoubtedly have a major impact on how we vision the intake of selenium in the diet of humans and other mammals. It has been known for sometime that diets containing sufficient or supplemental amounts of selenium have beneficial effects in preventing certain forms of cancer possibly through the action of enriching the selenoprotein population [1]. However, caution should be expressed in that once a malignancy is initiated, then adequate or enriched amounts of selenium in the diet may serve to drive tumorigenesis. Our study adds an important layer towards understanding the roles of redox processes in cancer developments and the use of diet in cancer prevention and treatment.

## Materials and Methods

### Materials

^75^Se (specific activity 1000 Ci/mmol) was obtained from the Research Reactor Facility, University of Missouri, Columbia, MO. PVDF membrane, NuPage 4–12% Bis-Tris gels, hygromycin B, lipofectamine 2000, Dulbecco's modified Eagle's medium (DMEM), antibiotic-antimycotic solution and fetal bovine serum were from Invitrogen Life Technologies. siRNA vector pSilencer 2.1-U6 Hygro was purchased from Ambion, Inc. and ρ-iodonitrotetrazolium violet (INT) from Sigma. Antibodies against DNA polymerase α, β, δ and ε were purchased from Abcam, Inc. and antibodies against PCNA and Cdc45 were from Santa Cruz Biotechnology, Inc.. BCA protein assay reagent, SuperSignal West Dura Extended Duration Substrate and HRP-conjugated secondary antibody were from Thermo Fisher Scientific Inc. FITC BrdU flow kit was purchased from BD Pharmingen™. Cancer cell line DT which encodes oncogenic *k-ras* and originated in NIH3T3 (parental) cells was generously provided by Dr. Yoon Sang Cho-Chung and NIH3T3 cells were obtained from the American Type Culture Collection. LLC1 (mouse Lewis lung carcinoma) cells were obtained as given [16] and human cell lines ACHN (human kidney renal cell carcinoma), A549 (human lung non-small cell carcinoma), HCT116 (human colon cell adenocarcinoma) and SNB19 (human cell glioblastoma) were obtained from the NCI, DTP, DCTD Tumor Repository at the NIH, Bethesda, MD.

### Culture of mammalian cells and cell growth assays

NIH3T3 and DT cells were grown in DMEM supplemented with 10% fetal bovine serum and antibiotic-antimycotic solution at 37°C, 5% CO_2_ in a humidified incubator. Stably transfected siTR1 (TR1 knockdown) DT cells and stably transfected pU6-m3 control DT cells were prepared by transfecting with the corresponding constructs with lipofectamine 2000 and then selecting cells in the presence of 500 µg/ml of hygromycin B exactly as described [16].

Morphology of NIH3T3, DT, DT/pU6-m3 and DT/siTR1 cells were assessed during exponential growth by seeding cells onto 60 mm culture dishes, the cells grown exponentially and photographed with an inverted phase-contrast microscope. Growth rates of NIH3T3, DT/pU6-m3 and DT/siTR1 cells were assessed by seeding 2×10^5 ^cells/60 mm culture dish and after 48 hrs growth, cells were harvested with trypsin-EDTA, and the cells counted by the trypan blue extrusion method [16]. For assessing the growth rate of cells in serum-deficient condition, cells (5×10^5 ^cells/60 mm culture dish) were seeded and harvested after 24 and 48 hours incubation in medium containing all components as complete medium except 0.5% FBS in place of 10% FBS, and the cells counted as above.

### Western blot analysis and ^75^Se labeling of cells

Techniques for western blot analysis and labeling of cells with ^75^Se have been detailed elsewhere [16]. Briefly, for western blot analysis, cells were washed with cold PBS and whole cell lysates prepared using lysis buffer (20 mM Tris-Cl, 150 mM NaCl, 1% Triton X-100, 0.5% sodium deoxycholate, 10 mM NaF, 5 mM EDTA, and proteinase inhibitor cocktail). The amounts of protein in cell extracts were measured using the BCA protein assay reagent, 30 µg of protein samples electrophoresed on NuPAGE 4-12% Bis-Tris gels, the separated proteins transferred to a PVDF membrane, and then incubated initially with primary antibody (polyclonal anti-TR1, anti-DNA Pol α, β, δ, ε, anti-PCNA and anti-Cdc45) and finally with HRP-conjugated secondary antibody. Membranes were reacted with SuperSignal West Dura Extended Duration Substrate and exposed to X-ray film.

For ^75^Se-labeling, cells were seeded onto a 6 well plate (3×10^5 ^cells/well), incubated 24 hours, then labeled with 40 µCi of ^75^Se for 24 hours, harvested and lysed as described above. 40 µg of each sample were applied to NuPAGE 4-12% Bis-Tris gel, electrophoresed, proteins stained with Coomassie Blue staining solution, the gel dried and exposed to a PhosphorImager (Molecular Dynamics). ^75^Se-Labeled selenoproteins on exposed gels were identified by autoradiography [16].

### Soft agar assay

Growth of cells in soft agar to assess their ability to sustain anchorage independent growth has been detailed elsewhere [16]. Briefly, a total of 1000 control, DT, DT/pU6-m3 or DT/siTR1 cells were suspended in 3ml of 0.35% noble agar in growth medium with 10% FBS, and spread evenly onto 60 mm plates covered with a 4 ml basal layer of 0.7% noble agar in DMEM. Plates were incubated in a humidified CO_2_ incubator for 14 days, 0.5 ml of fresh growth medium added onto the agar plate every 5 days. The colonies that developed were visualized by staining with ρ-iodonitrotetrazolium violet (INT) overnight and the plates photographed.

### Cell cycle analysis

Cells were seeded onto cell culture dish (5×10^5 ^cells/60 mm dish) and incubated overnight before transferring to serum-deficient media (0.5% FBS in DMEM). Cells were incubated with 5-bromo-2-deoxyuridine (BrdU) 6 hrs before harvesting, BrdU labeled cells harvested at determined time points (0 hr, 24 hrs and 48 hours) and stained with antibodies to monitor replicated DNA. Harvested cells were fixed and permeabilized with BD Cytofix/CytopermTM Fixation/Permeabilization solution for intracellular staining. For replicated DNA staining, incorporated BrdU was probed with anti-BrdU antibody and whole genomic DNA was stained with 7-amino-actinomycin D (7-AAD) following the manufacturer's procedures. Cells containing the respective DNA states were analyzed by flow cytometry using a FACS Calibur 2 Sorter (Beckton Dickinson) and the number of cells in each phase of the cell cycle quantitated by FlowJo (Tree Star, Inc.).

## Supporting Information

Text S1 (0.03 MB DOC)

Figure S1 (3.54 MB TIF)

Figure S2 (0.82 MB TIF)
