# Supplementary material for: Targeting Thioredoxin Reductase 1 Reduction in Cancer Cells Inhibits Self-Sufficient Growth and DNA Replication
Source: PLoS One. 2007 Oct 31;2(10):e1112. doi: 10.1371/journal.pone.0001112 (PMC2040202; doi:10.1371/journal.pone.0001112)
Supplement: Text S1 — (0.03 MB DOC) [file pone.0001112.s001.doc]

**Text S1**

The TR1 knockdown construct and the corresponding pU6-m3 control construct were prepared as given in the Supplementary Methods. The targeting site of human siTR1 had similar homology and was located in a similar region in the 3’-untranslated region (3’-UTR) as that in mice [S1]. A549 (human lung non-small cell carcinoma) and HCT116 (human colon cell adenocarcinoma) cells were stably transfected with pU6-m3 and siTR1 constructs and cell extracts analyzed for TR1 expression by western blotting (Fig. S1, left 2 panels). TR1 was effectively knocked down by the siRNA in both human cell lines.

The growth properties of HCT116 cells transfected with siRNA and control constructs in soft agar were examined (Fig. S1, right panel). HCT116 cells transfected with siRNA grew poorly in soft agar while the corresponding cells transfected with the control construct grew more efficiently. Similar effects were observed in A549 cells (data not shown). These data further establish that the growth properties of the TR1 knockdown cells were more similar to those expected of wild type cells compared to the control cancer cells, providing further evidence that reduction in TR1 levels uniquely affects growth properties of cancer cells.

Reduced levels of thioredoxin have been shown to prolong the S phase by reducing the dNTP pool sizes [S2,S3]. As this could possibly also be a reason to account for the retarded growth rate of DT/siTR1 cells in serum-deficient medium, we examined the dNTP pool sizes in control (NIH3T3), DT/pU6-m3 and DT/siTR1 cells grown in serum-deficient medium (Fig. S2). There appeared to be a loss in each dNTP, and particularly in dTTP and dCTP in control cells and possibly in dTTP in DT/pU6-m3 cells, over the growth period. However, there did not appear to be any significant loss in dNTP pool sizes in DT/siTR1 cells to account for its reduced growth rate. Therefore, dNTP pool size was apparently not a factor that could explain the consequences of TR1 knockdown in cancer cells.

**Supplementary Methods**

**Materials.** All materials used in obtaining the data in the Supplementary Methods were the same at those given in the Materials of the full manuscript with the exception of those used in carrying out the analysis of dNTP pools. dATP, dTTP, dGTP and dCTP standards were purchased from Invitrogen Life Technologies, DE81 cellulose membranes were from Whatman® and DNA Polymerase I, Large (Klenow) Fragment is from New England Biolabs Inc. Primers for polymerase reaction were synthesized by Sigma Genosys.

**Knockdown of TR1 in human cancer cells.** The 3’-UTR of human TR1 mRNA (accession number: S79851) was surveyed using the diDESIGN program (Dharmacon, Inc.) to select potential targeting sites for knocking down TR1 expression. The targeting site selected encoded ttattctcgttgtcaagtt, nucleotides 2273-2291. Sense-antisense oligonucleotides for TR1 knockdown were annealed and inserted into the *BamH*I-*Hind*III cloning sites in pU6-m3 according to instructions detailed in the cloning manual of the pSilencer 2.1U6 Hygro vector. The resulting constructs, designated siTR1 (targeting construct) or pU6-m3 (control or empty vector containing the same construct as siTR1 except lacking the siRNA), were confirmed by sequencing [S1,S4].

A**CKNOWLEDGEMENTS**

The authors appreciate the insights and advice on carrying out the analysis of the dNT pools provided by Drs. Gary F. Merrill, Christopher K. Mathews and Linda J. Benson.

**Supplementary Figure Legends**

**Figure S1** Targeted knockdown of TR1 expression in human cancer cells and growth in soft agar. (A) A549 (human lung non-small cell carcinoma) and HCT116 (human colon cell adenocarcinoma) were stably transfected with the pU6-m3 (control) vector or the siTR1 knockdown vector and TR1 expression examined by western blotting. (B) Examination of anchorage independent growth of HCT116/pU6-m3 and HCT116/siTR1 by soft agar assay. Details of the experiments shown in the figure are given in Methods and the text.

**Figure S2** dNTP pools in wild type and stably transfected DT cells. Control (NIH3T3, parental cells), DT/pU6-m3 and DT/siTR1 cells were grown in serum-deficient medium, the cells harvested at the time points shown and counted. 1.5×10^6^ cells were taken of each cell line, the cells collected by centrifugation and dNTPs extracted, dissolved in 120 μl of deionized water and dNTP levels determined by the DNA polymerase base enzymatic method [S2,S3].

**Supplementary References**

1. Yoo MH, Xu XM, Carlson BA, Gladyshev VN, Hatfield DL (2006) Thioredoxin reductase 1 deficiency reverses tumor phenotype and tumorigenicity of lung carcinoma cells. [J Biol Chem](javascript:AL_get(this, 'jour', 'J Biol Chem.');) **281:** 13005-13008.

2. Song S, Pursell ZF, Copeland WC, Longley MJ, Kunkel TA, et al. (2005) DNA precursor asymmetries in mammalian tissue mitochondria and possible contribution to mutagenesis through reduced replication fidelity. Proc Natl Acad Sci U S A **102:** 4990–4995.

3. Koc A, Mathews CK, Wheeler LJ, Gross MK, Merrill GF (2006) Thioredoxin is required for deoxyribonucleotide pool maintenance during S phase. J Biol Chem **281:** 15058 – 15063.

4. Xu XM, Mix H, Carlson BA, Grabowski PJ, Gladyshev VN, et al. (2005) Evidence for direct roles of two additional factors, SECp43 and Soluble Liver Antigen, in the selenoprotein synthesis machinery. J Biol Chem **280:** 41568–41575.
