# Supplementary figures and images for: Targeting Thioredoxin Reductase 1 Reduction in Cancer Cells Inhibits Self-Sufficient Growth and DNA Replication
Source: PLoS One. 2007 Oct 31;2(10):e1112. doi: 10.1371/journal.pone.0001112 (PMC2040202; doi:10.1371/journal.pone.0001112)

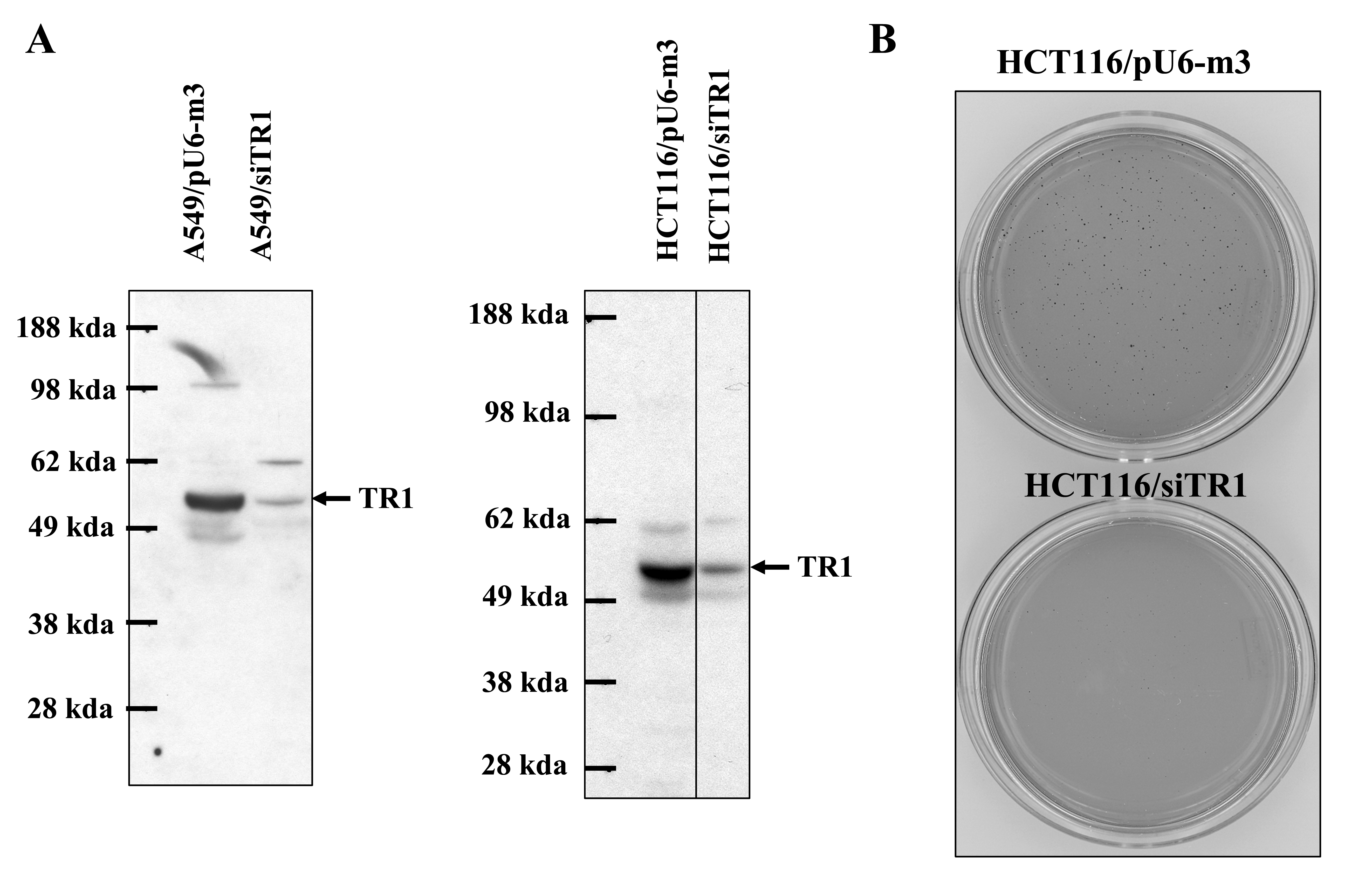

Supplement: Figure S1 — (3.54 MB TIF) [file pone.0001112.s002.tif]
